# Feline Leishmaniosis: A Retrospective Study of Seroprevalence in Cats in the Campania Region, Southern Italy

**DOI:** 10.3390/ani15121801

**Published:** 2025-06-18

**Authors:** Laura Cortese, Giulia Abate, Pasquale Santoro, Elvira Improda, Gianmarco Ferrara, Vincenzo Lucidi, Antonio Sica, Giuseppe Iovane, Serena Montagnaro

**Affiliations:** 1 Department of Veterinary Medicine and Animal Productions, University of Naples, “Federico II”, Via Delpino 1, 80137 Naples, Italy; giulia.abate.1995@virgilio.it (G.A.); elviraviolaimproda@gmail.com (E.I.); antonio.sica@unina.it (A.S.); iovane@unina.it (G.I.); 2 Department of Paediatrics and Laboratory Medicine, University of Naples, “Federico II”, Via Sergio Pansini 5 80134 Napoli, Italy; pasantor@unina.it; 3 Department of Veterinary Science, University of Messina, Polo Universitario dell’ Annunziata, Viale G. Palatucci 23, 98168 Messina, Italy; gianmarco.ferrara1@unime.it; 4 Independent Researcher, 63072 Castignano, Italy; lucidivin@gmail.com

**Keywords:** *Leishmania infantum*, cats, seroprevalence, Italy, feline retrovirus, risk factors, indirect fluorescent antibody technique

## Abstract

Feline leishmaniasis (FeL), caused by *Leishmania infantum*, is increasingly reported in areas of endemic Mediterranean canine leishmaniasis (CanL), making it an emerging feline disease. This cross-sectional study investigated *L. infantum* seroprevalence and risk factors in 229 domestic cats from the Campania region of southern Italy, a CanL endemic area, between January 2023 and December 2024. Serum samples were tested for *L. infantum* antibodies (IFAT) and for FIV/FeLV. Seropositivity (IFAT titre ≥ 1:40) for FeL was detected in 12/229 (5.2%) of the cats tested. No statistically significant correlation was found between seropositivity for *L. infantum* and the variables considered. However, outdoor cats and FIV/FeLV-seropositive cats had higher prevalence rates: 10.6% and 7.4%, respectively. Of the 12 seropositive cats, 7 (58.3%) had an antibody titre of 1:40, 2 (16.6%) of 1:80 and 3 (25.0%) a titre of 1:160. Of the 12 cats positive for FeL, 2 (16.6%) were also positive for FIV. Our results confirm the exposure to *L. infantum* and the serological response in cats from southern Italy. The low prevalence could be due to owners using mosquito control products in the household that would also protect cats. Further investigation is essential to clarify risk factors and improve our understanding of the epidemiology of FeL in this endemic area.

## Simple Summary

A retrospective study on the presence of antibodies against *Leishmania infantum* in owned cats in the Campania region was conducted from January 2023 to December 2024. Serum samples from cats (*n* = 229) were tested for antibodies against *L. infantum* using the immunofluorescent antibody test (IFAT) and for feline immunodeficiency virus (FIV) and feline leukaemia virus (FeLV) using a commercial rapid test kit. Of the 12 seropositive cats, 7 (58.3%) had an antibody titre of 1:40, 2 (16.6%) of 1:80 and 3 (25.0%) a titre of 1:160. Of the 12 cats that were positive for *L. infantum*, 2 (16.6%) were also positive for FIV.

## 1. Introduction

Leishmaniasis, a vector-borne zoonosis caused by protozoan parasites of the genus *Leishmania*, is a global health problem. It affects both humans and a variety of animal species through the bite of phlebotomine sandflies [1]. *Leishmania infantum* (*Kinetoplastida: Trypanosomatidae*) is the most widespread species in Italy and the countries of the Mediterranean Basin [2]. Consequently, the geographical distribution, the infection patterns of animals and the infection patterns of humans with *L. infantum* are closely linked to the distribution of the vectors [3]. While dogs are recognised as the primary reservoir host for *L. infantum* and play a crucial role in disease transmission in endemic areas such as Italy, the involvement of other domestic and wild animals, including cats, in the epidemiological cycle is increasingly recognised [4,5].

In regions where canine leishmaniasis (CanL) is endemic, cats are naturally exposed to *L. infantum*. Although feline leishmaniasis (FeL) has historically been considered a low-incidence disease, recent decades have seen a worrying increase in reported cases of *L. infantum* infection in cats, not only in endemic areas but also sporadically in non-endemic regions [6]. While infected cats often remain clinically asymptomatic, the presence of co-infection with feline retroviruses such as FIV and/or FeLV has been associated with the development of clinical signs [7], although the exact role of these retroviruses in the pathogenesis of FeL remains a matter of debate.

Despite advances in the understanding of FeL, challenges in diagnosis and treatment remain due to the variable clinical presentations and complexity of laboratory diagnosis [6,8,9]. Indeed, the manifestations of the disease are variable and include non-specific signs such as lymphadenopathy, weight loss, pallor of the mucous membranes, ocular and dermatological manifestations (ulcers and nodules on the head or pelvic limbs are common dermatological signs in cats and atypical presentations are more frequent in cats than in dogs) as well as haematological abnormalities such as hyperglobulinaemia and non-regenerative anaemia [9,10,11]. In addition, the occurrence of subclinical infections requires the use of specific direct and indirect diagnostic tests, making it difficult to determine the exact aetiology [2,10]. Serological methods such as the immunofluorescent antibody test (IFAT) and the enzyme-linked immunosorbent assay (ELISA) are standard for FeL diagnosis and research, although there is evidence that the IFAT is more sensitive in early or subclinical infections, while the ELISA may be more suitable for clinically apparent disease [4,10,12,13].

Molecular techniques such as conventional PCR, nested PCR and real-time PCR have become basic diagnostic tools for the detection of Leishmania DNA in various samples from cats (e.g., whole blood, buffy coat, conjunctival and oral swabs, skin, nasal tissue). These methods, which target multicopy genes such as kinetoplast minicircle DNA (kDNA) or small subunit ribosomal DNA (SSU rDNA), are used in routine veterinary practise for the diagnosis of feline leishmaniasis (FeL) as well as in epidemiological studies of Leishmania infection in cats [10].

Some of these techniques, such as real-time PCR, have the added advantage of quantifying the parasitic DNA present in the sample. This quantitative capability makes them particularly useful for monitoring the efficacy of anti-Leishmania treatments.

However, it is important to emphasise that a positive PCR result only indicates the presence of parasite DNA at the time of sampling and does not allow the identification of previous infections. Such a result could indicate a transient infection rather than a full-blown disease. Therefore, molecular results must be interpreted with extreme caution, especially in a clinical context [10].

Italy’s diverse geographical and climatic landscape contributes to a high biodiversity and a significant prevalence of vector-borne diseases, including CanL, with regional differences in disease spread [14,15]. While previous Italian research on the ecopathology of FeL has often focused on wide geographical areas or specific northern and central regions, southern Italy, with the exception of the islands, is still underrepresented, despite the known endemicity of CanL in regions such as Campania [4,16,17,18,19,20,21,22].

Therefore, the aim of this study was to determine the seroprevalence of *L. infantum* infections in owned cats in the Campania region of southern Italy and to analyse the association between exposure to the parasite and factors such as age, gender and geographical location.

## 2. Materials and Methods

### 2.1. Ethical Approval and Consent to Participate

The study was approved by the Institutional Animal Welfare Committee of the University of Naples “Federico II” on 29 November 2022 (OPBA, CSV PG/2025/0029844, 7 March 2025). The animals were treated with respect for their welfare. Informed consent was obtained from the owners of the selected cats before the start of the study. All owners agreed that the data on age, gender, place of residence and lifestyle of the selected cats would be used for the risk factor analysis.

### 2.2. Study Area, Inclusion Criteria and Sample Size

The Campania region is located in southern Italy, stretches along the Tyrrhenian Sea and covers an area of 13,595 km^2^ (41°00′00″ N–14°30′00″ E) [23]. The climate is Mediterranean in the coastal areas and continental in the mountainous hinterland. The study was conducted among owned cats living in the study area. The sample size was calculated using the formula proposed by Thrusfield et al. (2018) [24] for a theoretically “infinite” population, adding the following information: expected prevalence of *L. infantum*: 12.0% [17], 95% confidence interval (CI) and desired absolute precision of 5%. The minimum sample size required was 162 cat serum samples.

The serum samples from 229 cats (exceeding the minimum sample size) were collected between January 2023 and December 2024 at the University Veterinary Teaching Hospital (Ospedale Veterinario Universitario Didattico—OVUD) of the Department of Veterinary Medicine and Animal Productions (University of Naples “Federico II”). Cats owned by clients were included in the study if they: (1) lived in the Campania region; (2) had undergone a blood test after a clinical visit; and (3) the cats had not received prophylactic treatment (repellents, collars, pipettes, etc.). For each sample, the age, gender, lifestyle (indoor/outdoor) and location of the animals were recorded. The animals were categorised into three age groups: kittens ≤1 year old, young adult >1–6 years old and senior >7 years old [25].

### 2.3. Sample Preparation and Serological Analysis

Blood samples were collected from each cat using a Vacutainer tube without anticoagulant and centrifuged immediately. During centrifugation, the samples were spun at 1300–1800× *g* for 20 min. The serum was then collected and stored at −20 °C prior to analysis.

The test procedure followed the protocol of the World Organisation for Animal Health (WOAH) [26]. In brief, the antigen was prepared as follows: parasites were collected and immediately centrifuged at 4 °C for 20 min at 300× *g*. The pellet was suspended in PBS for 10 min. The slides were washed three times and the parasites were suspended at a final concentration of 2 × 10^6^/mL. The suspension (20 µL) was spread on each well of a multispot glass slide (12 wells, 5 mm, Thermo Fisher Scientific, Waltham, MA, USA). Slides were dried in a vertical flow cabinet equipped with both HEPA and carbon filters. Promastigotes were fixed in cold acetone at −20 °C for 20 min, and stored at −25 °C for a maximum of 60 days [26].

Anti-Leishmania antibodies were detected using 20 μL of rabbit fluorescein isothiocyanate-conjugated anti-cat IgG diluted 1:40 (Sigma-Aldrich, Darmstadt, Germany). In brief, 20 µL of the appropriately diluted sera were added to each well and incubated at 37 °C for 30 min. The slides were washed three times in cold PBS for 10 min. Then, 20 μL of diluted (1:100) fluorescein isothiocyanate-conjugated anti-cat IgG with 0.0005% Evans blue (Sigma Chemical; St. Louis, MO, USA) was added to each well and the slides were again incubated at 37 °C for 30 min. Counterstaining was important to remove the non-specific background. After incubation, slides were again washed three times with cold PBS (10′) and mounted with 50% glycerol. Each sample was run in duplicate and read by two people with specialised experience in this field. The observation was performed at a magnification of 400–600×. There are no standardised IFAT cutoff values for cats and other domestic mammals [26], while the IFAT cutoff value for CanL ranges from 1/40 (indicating exposure but not necessarily confirmed infection) to 1/160 (indicating confirmed infection) [27]. In some studies conducted in Italy, the cutoff value was set at 1:80, in others at 1:40 [13,18,20,28]. In this study, all sera in which antibodies against *L. infantum* with a titre ≥1:40 were detected were considered compatible with subclinical infection and therefore positive [29]. Although there is limited data on the performance of IFAT for the diagnosis of FeL, in a study conducted in endemic and non-endemic areas for *L. infantum*, Persichetti et al. reported sensitivity and specificity values of 75% and 97%, respectively [13], with a cutoff of 1:80.

All 229 cats participating in the study were tested for FIV/FeLV to determine whether they were positive for these diseases, which can affect the animal’s immune status.

For FIV/FeLV diagnosis, a commercial rapid test kit (SNAP FIV/FeLV Combo Plus Test, IDEXX, Hoofddorp, The Netherlands) was used to detect antibodies against FIV and FeLV antigens. This test can detect the core protein of the FeLV virus (p27) and antibodies against several FIV proteins (p15, p24 and gp40) simultaneously. The results were determined by visual inspection. Sensitivity and specificity of the test are: FIV: 100% (95% IC: 93.1–100%) and 99.6% (95% CI: 98.5–99.9%), respectively; FeLV: 92.3% (95% confidence interval (CI): 79.7–97.3%) and 97.3% (95% CI: 95.5–98.4%), respectively [30].

### 2.4. Statistical Analysis

The statistical software MedCalc version 16.4.3 (MedCalc Software, Ostend, Belgium; 2016) was used to analyse the association between the dependent (positivity for *L. infantum* antibodies) and independent factors (FIV/FeLV positivity, place of residence, age, gender and lifestyle) using the chi-square statistic. Variables associated with seroprevalence for *L. infantum* were applied to binary logistic models using JMP Pro version 18.0.0 (SAS Institute Inc., Campus Drive, Cary, NC, USA). A *p*-value < 0.05 was considered significant. A goodness-of-fit test (GraphPad Prism 7.0 for Windows) was used to test the hypotheses for the statistical tests used. The strength of the association between the variables was quantified by calculating odds ratios (OR) and their 95% confidence intervals (95% CI) (JMP Pro version 18.0.0—SAS Institute Inc., Campus Drive, Cary, NC, USA).

## 3. Results

A total of 229 owned cats from Campania, southern Italy, were included in the present study. No obvious clinical and dermatological signs attributable to infection with *L. infantum* were detected during the examination. The cats were brought to the OVUD for veterinary examination or sterilisation. Information on age, gender, lifestyle, FIV/FeLV infection and geographical location was recorded for all samples.

The sample consisted of 140 male cats (61.0%), of which 33.0% were neutered, and 89 female cats (39.0%), of which 16.0% were neutered. The average age was 6.3 years old, 13.5% were kittens (≤1 year old), 44.5% were young adults (>1–6 years old) and 41.9% were seniors (>7 years old). All cats recruited for the study were tested for FIV and FeLV. A significant proportion of samples (79%) were collected in the province of Naples, while the provinces of Caserta, Avellino and Salerno accounted for 9%, 8% and 4% of samples, in that order.

**Figure 1:**
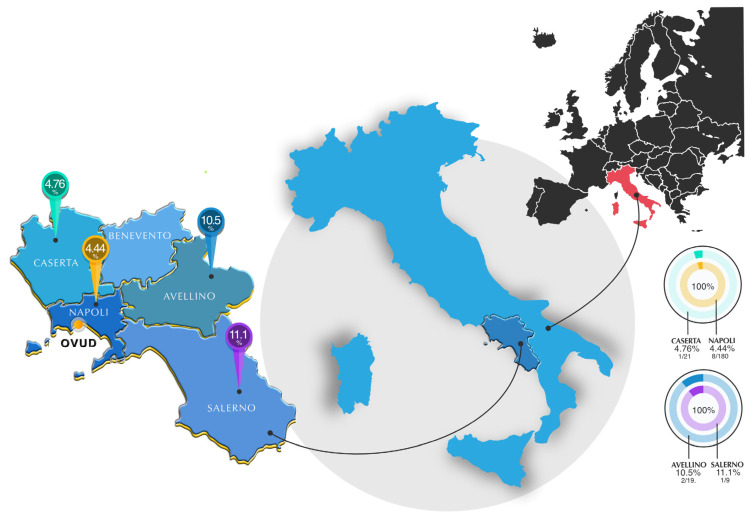
Map of cat sampling sites (*n* = 229) in the Campania region (Italy) showing *L. infantum* positivity rates by province and the location of the OVUD. The map was created with Affinity Designer 2, version 2.6.3.3322—Serif (Europe) Ltd. Nottingham (UK).

Seropositivity (IFAT titre ≥1:40) for L. infantum was detected in 5.2% (12/229, 95% CI: 2.4–8.1%) of the cats tested (Figure 1). Data analysis showed that there was no statistical correlation between FeL seropositivity and the variables considered.

To confirm the robustness of these results, a goodness-of-fit test was performed to further confirm the lack of correlation between FeL seropositivity and the variables considered. These results indicate that the variables considered do not in themselves represent an independent risk factor for feline leishmaniasis (*p* > 0.05).

However, among the variables analysed, cats with access to outdoor areas (10.6%, 95% CI: 1.8–19.5; OR: 2.976) and FIV and/or FeLV seropositivity (7.4, 95% CI: 0.00–17.3; OR: 1.266) had the highest prevalence rates, while the prevalence values were similar in the age groups considered and in males and females. The results are summarised in Table 1.

**Figure 2:**
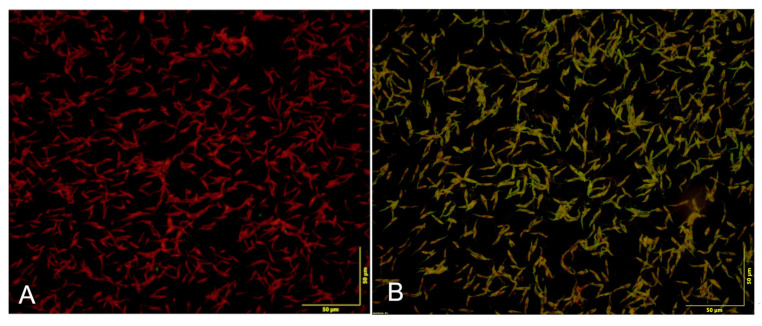
Indirect immunofluorescence antibody test. Cytoplasmic fluorescence of *Leishmania infantum* promastigotes. (**A**) Feline negative sample. (**B**) Feline positive sample.

Of the 12 seropositive cats, 7 (58.3%) had an antibody titre of 1:40, 2 (16.6%) of 1:80 and 3 (25.0%) a titre of 1:160 (Figure 2). Of the 12 cats that were positive for *L. infantum*, 2 (16.6%) were also positive for FIV.

## 4. Discussion

This study investigated the seroprevalence of *L. infantum* infections in domestic cats in the Campania region of southern Italy, an endemic area for canine leishmaniasis [21,22]. Although the overall prevalence of 5.2% (12/229; 95% CI: 2.4–8.1%) found in our study is relatively low, it confirms the presence of FeL in this region. It is noteworthy that all seropositive cats had no clear symptoms attributable to FeL, which emphasises how difficult it is to diagnose FeL based on clinical signs alone [2,9,10].

The prevalence of FeL varies greatly from country to country. For example, a seroprevalence of 15% to 22% has been reported in cats from the Mediterranean Basin, where *L. infantum* is endemic [2,31], while the seroprevalence in South America, particularly in Brazil, another endemic area, is much lower at around 4%, both in owned and unowned cats (e.g., in shelter or community-cared strays) [32,33,34].

In addition, a multicentre study involving six Mediterranean countries found an overall prevalence of FeL infection of 14.5% in owned cats [2]. When we compare the prevalence of FeL found in our study (5.2% 95% CI: 2.35–8.13%) with the prevalence found in other Mediterranean countries, we note that the value is lower than the positivity rates found in shelter/free-roaming cats in Spain (from 10.2 to 17.3%) [2,35,36], but similar to the value found in owned cats in the same country (7.2%) [2]. Higher seroprevalence rates were also found in owned cats in other Mediterranean countries, e.g., Greece (22.8%), Israel (16.2%), Portugal (33.3%), France (12.7%) and Turkey (15.6%) [2,31].

The results of our study indicate an FeL seroprevalence of 5.2%, a lower value compared to the 11% found by Carbonara et al. [2] in a study in the Mediterranean Basin. This difference probably reflects the different sampling strategies and inclusion criteria, such as a history of outdoor access. However, regional differences in the seroprevalence of FeL can also be observed in Italy. Studies have reported seroprevalence rates between 1.6% and 12.5% in northern Italy and between 2.3% and 4.3% in central Italy [3,4,16,18], while southern Italy and the islands of Sardinia and Sicily have higher prevalence rates of 10% and 11.8%, respectively [4,17,20].

The lack of statistically significant correlations between *L. infantum* seropositivity and the analysed variables (age, gender, lifestyle, FIV/FeLV co-infection and geographical location) is an interesting finding. While previous studies have pointed to possible risk factors such as access to the outdoors and co-infection with retroviruses [2,4,36], our data showed no clear correlation. However, it is worth noting that outdoor cats and FIV/FeLV-seropositive cats had the highest prevalence rates, indicating possible trends that should be further investigated with larger samples. The distribution of IFAT titres among the seropositive cats showed that the majority had low titres (1:40), while a smaller proportion had titres between 1:80 and 1:160. This observation is consistent with the non-specific nature of the symptoms caused by *Leishmania* in these animals and may reflect different phases of exposure or immune response [9,10,37]. This high variability of results once again highlights the lack of standardisation of the technique for detecting *Leishmania* infections in cats, which makes it very difficult to compare the results of different studies [2,10].

Moreover, this difficulty becomes even greater when comparing the performance of different tests, as there is no diagnostic gold standard. Despite the large number of published serological studies, very few studies have validated serological techniques by testing anti-Leishmania antibodies on a large number of serum samples from feline patients from endemic and non-endemic areas [13]. Therefore, a threshold of 1:40 was chosen to calculate prevalence, which is consistent with subclinical infection, as seroreactivity also indicates contact with the protozoan. In fact, serology is a measure of historical exposure and may or may not be associated with the presence of the pathogen at the time of sampling [15].

The detection of *L. infantum* seropositivity in cats from different provinces of the Campania region, with a concentration in the province of Naples, emphasises the widespread exposure to the parasite in this area. This finding is consistent with the known endemicity of canine leishmaniasis in the region and suggests that cats, although considered less susceptible, are exposed to the same vectors as dogs and could therefore represent a reservoir that is difficult to identify as it is often composed of asymptomatic and/or paucisymptomatic animals [4,7].

The co-infection of two *L. infantum*-positive cats with FIV emphasises the possible influence of retroviral infections on FeL. This observation emphasises the need for further studies to clarify the role of FIV and FeLV in the pathogenesis and clinical presentation of FeL. Indeed, the results reported in the literature on this topic are also contradictory, as evidenced by the fact that several studies have shown no statistical correlation between retrovirus infections and FeL positivity [33,38,39,40,41], while other studies have shown a statistical correlation with FIV but not between FeLV and FeL [4,19,41,42,43].

The low prevalence of 5.2% of FeL found in this study could be influenced by several factors. Apart from the methodological limitations and the presence of non-specific symptoms in FeL, it is important to consider the role of preventive measures taken by cat owners. Many pet owners, especially in endemic areas such as Campania, use domestic mosquito control products in their homes. These measures, such as mosquito nets, mosquito insecticides and mosquito repellents in the home environment, could significantly reduce the exposure of cats to leishmaniasis vectors, especially in animals that do not have access to the outdoors. The use of mosquito control products in the domestic environment may have helped to limit the transmission of the parasite to domestic cats, partially explaining the low prevalence observed. This hypothesis may be credible if we consider the growing awareness of leishmaniasis among pet owners and the availability of vector prevention products. Limitations of this study include the relatively small sample size, which may have limited our ability to detect statistically significant associations. In addition, the cross-sectional design of the study means that it is not possible to identify causal relationships.

Future longitudinal studies with larger cohorts and more comprehensive diagnostic approaches, including molecular diagnosis and parasitological examination, are needed to further investigate the epidemiology and clinical manifestations of FeL in the Campania region. This is particularly important considering the national epidemiological framework. In Italy, human zoonotic visceral leishmaniasis, caused by *L. infantum*, is endemic in all Tyrrhenian regions of the peninsula and on the islands. Campania (40–80 cases/year) and Sicily (30–50 cases/year) are currently the most affected regions [43]. The presence of such a pronounced endemicity in the Italian territory emphasises the importance of deepening the understanding of feline leishmaniasis, especially in high-incidence regions such as Campania.

## 5. Conclusions

To summarise, this study provides important data on the prevalence of FeL antibodies in domestic cats in southern Italy. Although the overall prevalence was low, the presence of seropositive cats, even without clinical signs, emphasises the need for increased awareness of FeL in endemic areas. Further research is needed to identify specific risk factors, standardise diagnostic approaches and better understand the pathogenesis of FeL in order to effectively control this infection, which is of both veterinary and public health importance.

## Figures and Tables

**Table 1:** Seroprevalence of FeL and analysis of risk factors according to age, gender, lifestyle, FIV/FeLV positivity and location in cats owned in the Campania region as determined by IFAT. SE: standard error; OR: odds ratio; CI: confidence interval.

Variable	*n*	Positive	%	SE % ^§^	95% CI ^¥^	χ^2^	*p*	OR ^#^	95% CI ^¥^
**Age**									
**Kitten ≤1 years old**	31	2	6.5	8.7	0.00–15.1			Ref. *	
**Young adult >1–6 years old**	102	5	4.9	4.2	0.7–9.1	0.115	0.944	1.337	0.184–6.572
**Senior >7 years old**	96	5	5.2	4.4	0.8–9.7			1.255	0.173–6.170
**Gender**									
**Male**	140	8	5.7	3.8	1.9–9.6	0.010	0.920	1.287	0.376–4.409
**Female**	89	4	4.5	4.3	0.2–8.8				
**Lifestyle**									
**Outdoor**	47	5	10.6	8.8	1.8–19.5	2.237	0.134	2.976	0.900–9.842
**Indoor**	182	7	3.9	7.8	1.1–6.6				
**FIV/FeLV infections**									
**Yes**	27	2	7.4	9.8	0.00–17.3	0.017	0.897	1.2667	0.267–5.991
**No**	202	12	5.9	3.3	2.7–9.2				
**Location**									
**Napoli**	180	8	4.4	3.0	1.4–7.5	1.933	0.586	Ref. *	
**Caserta**	21	1	4.7	9.1	0.00–13.9			0.930	0.158–17.70
**Avellino**	19	2	10.5	13.8	0.00–24.3			0.395	0.08–2.75
**Salerno**	9	1	11.1	20.5	0.00–31.6			0.372	0.05–7.32
**Total**	229	12	5.2	2.9	2.4–8.1		-		

^§^ SE: Standard error. ^¥^ 95% CI: 95% confidence interval. ^#^ OR: Odds ratio. * Ref: Reference category.

## Data Availability

Data is contained within the article.

## Abbreviations

The following abbreviations are used in this manuscript:

FeL	Feline leishmaniasis
CanL	Canine leishmaniasis
IFAT	Immunofluorescence antibody test
FIV	Feline immunodeficiency virus
FeLV	Feline leukaemia virus
ELISA	Enzyme-linked immunosorbent assay
PBS	Phosphate buffer saline
CI	Confidence interval
OVUD	University Veterinary Teaching Hospital
WOAH	World Organisation for Animal Health
OR	Odds ratio
